# Common Mental Health Conditions and Self-Stigma Among Saudi Male Adults: Implications for Promotion and Intervention

**DOI:** 10.7759/cureus.93452

**Published:** 2025-09-28

**Authors:** Mohammad A Jareebi, Ahmad Y Alqassim, Dhiyaa A Otayf, Mohammed A Najmi, Ali H Bakkarey, Eyad Z Omar, Mohammed H Hakami, Hadi A Hakami, Faisal M Jithmi, Saja A Almraysi, Alaa H Hakami, Sameer M Alqassimi, Mostafa N Mohrag, Ahmed A Bahri, Majed A Ryani

**Affiliations:** 1 Community and Family Medicine, Jazan University, Jazan, SAU; 2 Medicine, Jazan University, Jazan, SAU; 3 Psychiatry, Prince Sultan Military Medical City, Riyadh, SAU; 4 Internal Medicine, Jazan University, Jazan, SAU

**Keywords:** adult, anxiety, depression, male, mental illness, saudi arabia, self-stigma, stress

## Abstract

Background and objectives

Mental disorders, including depression, anxiety, and stress, are highly prevalent globally and pose significant public health challenges. In Saudi Arabia, sociocultural norms often lead to psychological distress and internalized stigma, discouraging men from seeking mental health care. This study aimed to assess the prevalence of depression, anxiety, stress, and self-stigma among Saudi males aged 18 years and above, and to examine their associations with sociodemographic characteristics.

Materials and methods

A cross-sectional study using convenience sampling was conducted among 612 male participants aged 18 years and above, residing in Saudi Arabia. Participants completed a 49-item online questionnaire covering sociodemographics, the Arabic version of the Depression, Anxiety, and Stress Scale-21 (DASS-21), and the Self-Stigma of Depression Scale (SSDS). Descriptive statistics, ANOVA, and multiple linear regression were used to analyze the data.

Results

The mean age of participants was 30 ± 12 years, with 68% residing in rural areas and 59% earning more than 10,000 Saudi Riyals (SAR) per month. Based on DASS-21 results, 312 (51%) of participants experienced depression, 306 (50%) had anxiety, and 220 (36%) reported stress. The mean overall self-stigma score was 52 ± 17, with 51% of participants categorized as having stigma. Regression analysis revealed that greater severity of depression, anxiety, and stress was associated with significantly lower self-stigma scores, indicating higher levels of internalized stigma. Conversely, married individuals demonstrated significantly lower levels of internalized stigma.

Conclusions

This study underscores the high prevalence of psychological distress and self-stigmatization among Saudi males. These findings highlight the need for gender-sensitive mental health interventions, stigma-reduction campaigns, and accessible community-based support systems tailored to the cultural context of Saudi Arabia.

## Introduction

Mental disorders are defined by the World Health Organization (WHO) as clinically significant disturbances in cognition, emotion regulation, behavior, or perception, typically associated with distress or impairment in functioning [1]. Among the various mental health conditions, depressive disorders, anxiety disorders, and chronic stress are among the most commonly diagnosed globally. According to recent WHO data, approximately 301 million people suffered from anxiety and around 280 million from depression in 2019 [1,2]. These conditions differ in their core features but are consistently associated with significant functional impairment, reduced quality of life, and increased healthcare utilization [3]. Depression is marked by prolonged low mood, loss of interest in activities, and disturbances in sleep or appetite, while anxiety disorders involve excessive fear and persistent worry that disrupt daily functioning. Although stress is often a normal and adaptive response to challenges, when it becomes chronic, it can cause serious psychological and physical consequences. Moreover, chronic stress frequently co-occurs with anxiety or depression, with comorbidity rates reaching up to 50% in some cases [1,2,4,5].

In Saudi Arabia, the burden of mental health conditions - particularly depression, anxiety, and stress - is considerable. A recent regional study found that 55% of participants reported depressive symptoms, 56% experienced anxiety, and 39% reported stress [6]. Despite the high prevalence, sociocultural norms in Saudi society often discourage men from seeking psychological support. Cultural expectations of emotional restraint, independence, and stoicism contribute to the stigmatization of mental illness, especially among males, who may perceive psychological distress as a sign of weakness or dishonor. These beliefs foster concealment of symptoms and reluctance to seek care, particularly when internalized stigma reinforces negative societal attitudes [7,8].

Self-stigma - defined as the internalization of negative societal attitudes and stereotypes toward mental illness - has profound consequences for mental health outcomes. It can erode self-esteem, foster feelings of shame, and act as a significant barrier to seeking timely psychiatric care [9]. This internalized stigma often leads to social withdrawal, unemployment, delayed help-seeking, and overall poorer prognosis [9-12]. One of the most severe consequences of delayed care due to self-stigma is suicide, particularly among men who are less likely to seek mental health services and more prone to use lethal means [13,14]. While women are more likely to report psychological symptoms, men tend to face greater stigma, especially in cultures where masculinity and emotional restraint are highly valued. In Saudi Arabia, sociocultural expectations around gender roles may further discourage men from expressing emotional vulnerability or accessing mental health support [7,8]. 

Given these considerations, this study had two primary objectives: (1) to assess the prevalence of depression, anxiety, stress, and self-stigma among Saudi males aged 18 years and above using validated scales; and (2) to examine their associations with key sociodemographic factors such as age, marital status, education, and income. In doing so, the study addresses a significant gap in gender-specific mental health data within the Saudi context, where cultural norms may distinctly shape men’s psychological experiences and help-seeking behaviors. As one of the few studies in Saudi Arabia to explore the relationship between psychological distress and self-stigma among men, it employed validated assessment tools to ensure accuracy and reliability. The findings are expected to inform culturally and gender-sensitive mental health policies and interventions tailored to the specific needs of Saudi males.

## Materials and methods

Study design and sample size

This study employed a cross-sectional design and a non-random convenience sampling method. The study population included Saudi individuals aged 18 years and older who provided informed consent. Individuals who did not meet the inclusion criteria were excluded. The sample size was calculated using an online tool (raosoft.com). With a 95% confidence interval (CI), a margin of error of 3.9%, and a distribution rate of 50%, the required sample size was 612 participants.

Data collection tool

The data collection tool was designed and developed based on relevant cultural and scientific variables and was updated and validated following a review of the current literature. Additionally, the tool was reviewed for cultural appropriateness and content validity by two experts: one psychiatrist and one public health/family medicine physician. They provided iterative feedback across multiple drafts until approval. The tool was subsequently piloted with a subset of participants, and minor revisions were made under expert supervision before final implementation. The tool consisted of 49 questions divided into three sections: sociodemographic characteristics, the Depression, Anxiety, and Stress Scale-21 (DASS-21), and the Self-Stigma of Depression Scale (SSDS).

The first section inquired about participants’ age, gender, employment status, educational level, monthly income, place of residence, and geographic region. The second section comprised the Arabic version of the DASS-21, a validated instrument used to assess common mental health conditions - namely, stress, anxiety, and depression. It comprises 21 items, divided into three subscales, and is structured using a four-point Likert scale, ranging from 0 (did not apply to me at all) to 3 (applied to me very much or most of the time). Scores on each subscale were summed up and then multiplied by two to yield a final score ranging from 0 to 42 per subscale. Severe levels were defined as scores above 20 for stress, 14 for anxiety, and 25 for depression. The DASS-21 has demonstrated strong psychometric properties, with reported Cronbach’s alpha coefficients of 0.94 for depression, 0.87 for anxiety, and 0.91 for stress. Internal consistency has also been reported at α = 0.888 for depression, 0.864 for anxiety, and 0.890 for stress, indicating good convergent and discriminant validity, high internal consistency, and reliability. The DASS-21 is publicly accessible for non-commercial academic purposes [14-18].

The third section included the validated Self-Stigma of Depression Scale (SSDS), which assesses the extent of individuals' self-stigmatizing beliefs related to having a mental illness. The SSDS contains 16 items across four subscales: shame, self-blame, social inadequacy, and help-seeking inhibition. Items are rated on a 5-point Likert scale, with 1 representing "strongly agree" and 5 representing "strongly disagree." Higher total scores indicate lower levels of self-stigma, as the items are coded accordingly. Permission to use the SSDS was obtained from the author of the validated Arabic translation [19,20]. The full questionnaire used in this study can be found in the appendix (Appendix 1).

Data collection process

The data collection process was carried out from June to December 2023. The questionnaire was distributed via social media platforms (WhatsApp, Twitter, Telegram, Facebook, and Snapchat). This approach benefited from the widespread use of social media in Saudi Arabia, facilitating the recruitment of diverse sociodemographic groups and minimizing barriers to participation.

All research assistants received standardized instructions covering survey administration, ethical conduct, and handling participant queries to ensure consistency in data collection procedures. Additionally, the research team regularly reviewed the dataset and promptly managed incomplete and duplicate responses. This approach, along with the use of a validated data collection tool, ensured the quality, reliability, and robustness of the collected data.

Statistical analysis

All data were analyzed using RStudio (version 4.2.3, R Foundation for Statistical Computing, Vienna, Austria). Mental health conditions (depression, anxiety, and stress), self-stigma, and sociodemographic characteristics were summarized using descriptive statistics, including means, standard deviations, and frequencies. Severity classifications of stress, anxiety, and depression were based on the established DASS-21 cut-off scores. Prevalence rates were calculated for mental health conditions and self-stigma. One-way analysis of variance (ANOVA) was conducted to assess differences in mean self-stigma scores across varying levels of mental health symptom severity. Multiple linear regression analysis was performed to identify associations between self-stigma and sociodemographic and mental health variables. Self-stigma was treated as the dependent variable. Independent variables included BMI, smoking status, age, place of residence, and mental health scores. Variance inflation factors (VIFs) were used to assess multicollinearity among predictors. All statistical tests were two-sided, with a significance level set at α = 0.05. When applicable, 95% confidence intervals (CIs) were reported. The STROBE guidelines were followed for reporting this observational study.

Ethical approval

The Standing Committee for Scientific Research at Jazan University approved this study (Reference No. REC-45/05/871, dated 04/12/2023). All procedures involving human participants were conducted in accordance with the ethical standards of the institutional and/or national research committee, the 1964 Helsinki Declaration and its later amendments, or comparable ethical standards. Participants were provided with a detailed explanation of the research project, its objectives, outcomes, and expected benefits.

## Results

Sociodemographic, habitual, and anthropometric characteristics

This study included 612 male participants with a mean age of 30 ± 12 years. Participants reported an average of 7 ± 3.7 siblings, indicating large family structures. The majority of participants were Saudis (600, 98%). Approximately two-thirds of participants (417, 68%) were from rural areas, while 195 (32%) were from urban areas. Most participants were single 374 (61%), while 238 (39%) were either married or previously married. Finally, 139 (23%) and 220 (36%) of participants earned between 10,000 and 14,999 and more than 15,000 Saudi Riyals (SAR) per month, respectively. Further details about the sample are provided in Table 1. Table 2 presents the behavioral and anthropometric characteristics of the participants. Participants had a mean BMI of 26 ± 6 kg/m² (classified as overweight based on WHO categorization of BMI) [19]. Regarding participants’ habitual characteristics, 150 (25%) reported currently smoking, with the majority (64, 10%) being cigarette smokers.

**Table 1:** Profile of Study Participants: Sociodemographic Overview (n = 612) SD: standard deviation, SAR: Saudi Riyal

Characteristics	Mean ± SD
Age	30 ± 12 years
Family members	7 ± 3.7 kids/household
Characteristics	Frequency (%)
Nationality	
Saudi	600 (98%)
Non-Saudi	12 (2%)
Residence	
Rural	417 (68%)
Urban	195 (32%)
Marital status	
Single	374 (61%)
Married	230 (38%)
Divorced/widowed	8 (1%)
Income, SAR	
Less than 5,000	147 (24%)
5,000-9,999	106 (17%)
10,000-14,999	139 (23%)
≥15,000	220 (36%)

**Table 2:** Behavioral and Anthropometric Traits (n = 612) SD: standard deviation, BMI: body mass index

Characteristics	Mean ± SD
BMI	26 ± 6 kg/m^2^
Characteristics	Frequency (%)
Smoking status	
No	462 (75%)
Yes	150 (25%)
Smoking types (n = 150)	
Cigarettes	64 (10%)
Shisha	53 (9%)
Vape	33 (5%)

Depression, Anxiety, and Stress Scale-21 (DASS-21)

**Figure 1:**
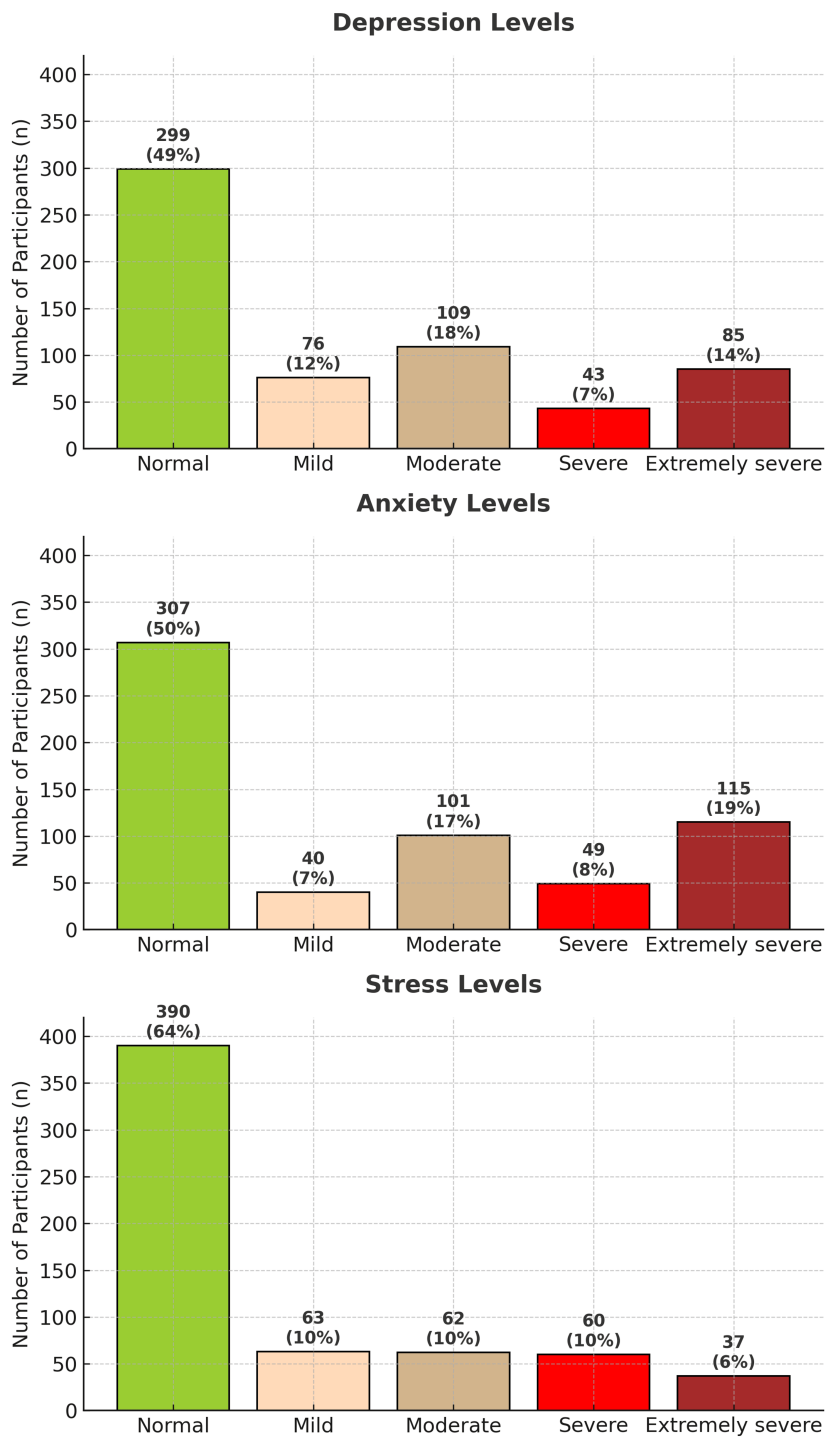
Depression, anxiety, and stress among study participants Based on the DASS-21 questionnaire [14]. The DASS-21 is publicly accessible for non-commercial academic purposes DASS-21: Depression, Anxiety, and Stress Scale-21

The levels of depression, anxiety, and stress obtained from the DASS-21 questionnaire are illustrated in Figure 1. The distribution of depression and anxiety levels appeared to be uniform, with half of the participants reporting no depression (299, 49%) or anxiety (307, 50%), while the other half reported varying degrees of depression and anxiety, ranging from mild to extremely severe. Similarly, approximately 390 (64%) participants experienced no stress, while 222 (36%) reported various levels of stress. The distribution of participants' details is depicted in Figure 1.

Self-stigmatization (outcome variable)

The findings of the self-stigmatization score from the SDSS questionnaire [18,19] are presented in Table 3. A higher score indicates lower stigmatization (i.e., a good score). The score is based on four domains used to conceptualize internalized stigma among the 612 male participants. These domains include shame score (mean = 14 ± 4.7), self-blame (mean = 11 ± 5.4), social inadequacy (mean = 14 ± 5), and help-seeking inhibition (mean = 14 ± 5.4). The overall stigma score, which is the sum of scores for all domains, was 52 ± 17. To better understand self-stigmatization, the overall stigma score was categorized based on a median value of 51, resulting in 312 (51%) of the participants labeled as "having stigma," while 300 (49%) were labeled as "not having stigma”.

**Table 3:** Self-stigmatization variables* (n = 612) ^*^Based on the SSDS questionnaire [19,20] (permission to use the SSDS was obtained from the author of the validated Arabic translation). ^**^Categorized based on mean value (51) SD: Standard deviation; SSDS: Self-Stigma of Depression Scale

Characteristics	Mean ± SD
Shame score	14 ± 4.7
Self-blame score	11 ± 5.4
Social inadequacy score	14 ± 5
Help-seeking inhibition	14 ± 5.4
Overall stigma score	52 ± 17
Characteristics	Frequency (%)
Self-stigmatization categories^**^	
Has stigma	312 (51%)
No stigma	300 (49%)

Self-stigma and DASS

**Figure 2:**
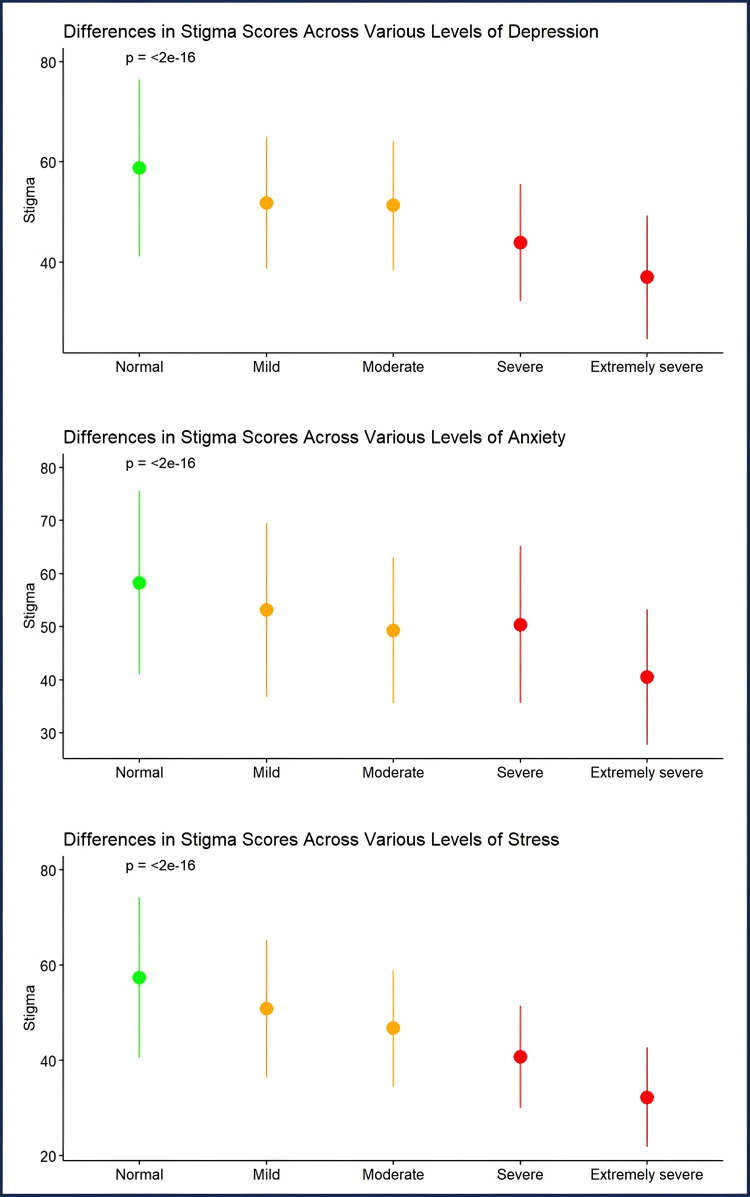
Stigma score difference among depression, anxiety, and stress levels

The analysis of the stigma scores across different levels of DAS variables revealed significant differences. The mean self-stigma score was the highest (i.e., indicating no stigma) among individuals with normal DAS levels across all variables (p<0.001). Additionally, the mean stigma score decreased as the severity of mental health conditions increased (i.e., extremely severe < severe < moderate < mild; p<0.001 for all DAS variables) (Figure 2)

Association between DASS variables and self-stigmatization

The relationship between mental health characteristics and self-stigmatization was further examined using a multiple linear regression analysis. The regression model included predictors such as age, gender, nationality, place of residence, BMI, smoking status, and the severity of depression, anxiety, and stress. Notably, the results indicated that greater severity of depression, anxiety, and stress, as well as residing in an urban area (β = -2.48, p = 0.071), were associated with lower self-stigma scores. Specifically, self-stigma scores decreased significantly among participants with higher levels of psychological distress (depression: mild β = -3.38, p = 0.121; moderate β = -3.33, p = 0.114; severe β = -6.98, p = 0.027; extremely severe β = -8.96, p = 0.005; anxiety: extremely severe β = -5.16, p = 0.054; stress: mild β = -2.85, p = 0.213; moderate β = -4.79, p = 0.070; severe β = -7.68, p = 0.013; extremely severe β = -13.77, p = 0.001). These findings indicate that individuals experiencing more severe mental health symptoms tend to report higher levels of self-stigma (Table 4).

**Table 4:** Association of DAS variables with self-stigma score: multiple linear regression CI: confidence interval; BMI: body mass index

Predictors	Self-stigma score		
	Beta	CI	p
Age	-0.13	-0.32 – 0.06	0.179
Nationality (reference: non-Saudi)			
Saudi	3.61	-12.89 – 5.67	0.445
Residence (reference: rural)			
Urban	-2.48	-5.18 – 0.22	0.071
Social status (reference: single)			
Married	4.94	0.22 – 9.66	0.04
Divorced	-1.38	-12.20 – 9.44	0.802
BMI	-0.07	-0.28 – 0.14	0.527
Smoking (reference: non-smoker)			
Yes	1.86	-2.21 – 5.93	0.37
Depression (reference: normal)			
Mild	-3.38	-7.66 – 0.89	0.121
Moderate	-3.33	-7.45 – 0.80	0.114
Severe	-6.98	-13.15 – -0.80	0.027
Extremely severe	-8.96	-15.27 – -2.66	0.005
Anxiety (reference: normal)			
Mild	-2.59	-7.81 – 2.63	0.33
Moderate	-4.68	-8.64 – -0.72	0.021
Severe	-1.67	-6.92 – 3.58	0.533
Extremely severe	-5.16	-10.40 – 0.08	0.054
Stress (reference: normal)			
Mild	-2.85	-7.33 – 1.63	0.213
Moderate	-4.79	-9.98 – 0.39	0.07
Severe	-7.68	-13.74 – -1.61	0.013
Extremely severe	-13.77	-22.17 – -5.36	0.001
Observations	612		

## Discussion

Consistent with national data, our findings highlight that Saudi males experience high levels of psychological distress, particularly depression and anxiety, coupled with substantial self-stigma. This co-occurrence suggests a syndemic pattern, where distress and stigma reinforce one another, potentially exacerbating barriers to care. Importantly, marital status emerged as a protective factor against stigma, while greater severity of mental health symptoms predicted significantly higher self-stigma levels. These results underscore the urgent need for culturally tailored interventions that address both psychological distress and stigma simultaneously.

These findings underscore a critical and growing public health concern: adult Saudi males are experiencing notably high rates of depression, anxiety, and stress [6]. Early detection and timely intervention within primary care systems are essential, particularly for high-risk cohorts who frequently delay treatment due to stigma and prevailing social norms [21]. In Saudi culture, expectations of emotional restraint and self-reliance exacerbate psychological distress and impede help-seeking, making stigma both a psychological and systemic barrier [8]. Moreover, self-stigma may intensify existing barriers in the Saudi healthcare system, including limited psychiatric resources, reliance on family support, and fear of judgment within tightly-knit communities. Addressing these challenges requires gender-sensitive, culturally attuned interventions such as public awareness campaigns, mental health literacy initiatives, and confidential service delivery models.

By focusing specifically on male mental health in the Saudi context, this study contributes to bridging gaps in community-based research. Evidence from Saudi settings indicates that awareness campaigns, educational programs, and confidential services can improve attitudes toward help-seeking and reduce self-stigma [22]. Tackling the persistent issue of stigma among Saudi males should remain a public health priority, with the overarching goal of reducing internalized shame and fostering help-seeking behaviors through targeted support and education initiatives.

The sample revealed that 51%, 50%, and 36% of the participants suffer from varying degrees of depression, anxiety, and stress, respectively. When comparing our findings with the available literature, we noted both similarities and discrepancies. Globally, a meta-analysis of multinational studies found that 3% of men suffer from major depressive disorder (MDD), 4% from generalized anxiety disorder (GAD), and 33.6% from stress [23-25]. Nationally, a systematic review and meta-analysis reported that 32.7% of adult males experience varying levels of depression [26]. Additionally, a national survey found that 11.4% of Saudi men are at risk of MDD, and 10.9% are at risk of GAD [4]. Furthermore, two studies conducted during the COVID-19 era reported depression, anxiety, and stress rates ranging from 25-33.5%, 8.3-13.5%, and 14.2-19.4%, respectively [27].

Another study on male adolescent students in Abha, Saudi Arabia, reported lower rates of depression (39.4%) but higher rates of anxiety (64.6%) and stress (57%) compared to our results [21]. These variations may stem from differences in sample size, age demographics, and assessment tools. Our study’s higher prevalence may also reflect the sensitivity of the DASS-21 in detecting even mild symptoms, as well as our recruitment strategy, which reached individuals who might otherwise underreport due to stigma. Importantly, the comparison of self-stigma rates between our study and global findings should be interpreted cautiously, as the representation differs between clinically diagnosed populations and community-based samples like ours, with Saudi sociocultural norms and masculinity expectations likely amplifying stigma among men.

Utilizing the SDSS, we found that 51% of participants experienced self-stigma. Globally, a systematic review and meta-analysis investigating the rates of depression-related self-stigma reported a prevalence of 29%, with married individuals having lower self-stigma, similar to our finding [28]. Another systematic review and meta-analysis reported self-stigma rates of 31.3% among individuals with serious mental illness, with rates increasing to 39% in the Middle East [29]. Nationally, our findings align with a study from Saudi Arabia, which reported significantly greater stigma among males compared to females (p = 0.006). However, divorced/widowed participants experienced less stigma than married and single individuals (p = 0.017) [8].

Additionally, a study in Abha, Saudi Arabia, highlighted gender disparities in perceived stigma, with males facing more stigma than females (62.6% vs. 59.9%). Notably, the study found that marital status - specifically, widowed participants - was the only significant factor affecting stigma levels, aligning with our findings on the importance of marital status [30]. Another study found that being male and having low knowledge of mental illness are risk factors for both self-stigma and public stigma [31]. These results underscore the high level of stigmatization and stereotyping faced by individuals with mental health conditions. While risk factors may vary across individuals and settings, this does not diminish the seriousness of the issue or the urgent need for effective management.

Self-stigma is characterized by feelings of shame, self-blame, social inadequacy, and help-seeking inhibition, which are evident in 51% of our participants. One study linked prejudice to lower self-perceived warmth and competence, which increases the likelihood of self-stigma [32]. This can, in turn, lead to increased psychological distress, both active and passive self-harm, and hinder access to healthcare [8,22,32]. Early identification and appropriate management can help prevent and mitigate these consequences.

Strengths and limitations

This study has several strengths that contribute to the robustness of our research. First, it adopted a comprehensive approach to assess the prevalence of depression, anxiety, stress, and self-stigmatization among adult males, providing a holistic understanding of mental health conditions in the region. Moreover, the study was guided by clearly defined objectives, offering direction for investigating mental health conditions, assessing self-stigma levels, and exploring the relationship between sociodemographic characteristics and mental health outcomes. The large sample size (612 participants) also enhanced the statistical power and the odds of detecting meaningful associations. Importantly, the use of validated Arabic versions of the DASS-21 and SSDS scales ensured cultural appropriateness and strengthened the reliability of measurement. These tools are widely recognized in mental health research and enable standardized, comparable assessment of depression, anxiety, stress, and self-stigma in the Saudi context.

However, several limitations must be acknowledged. First, the use of a convenience sampling method may introduce selection bias, although the widespread use of social media across all age groups in Saudi Arabia helped mitigate this risk. Cultural factors unique to Saudi Arabia may also influence the prevalence and severity of mental health conditions, limiting the applicability of the results to other regions or countries. Moreover, relying on self-reported data collected via online questionnaires introduces the possibility of response bias, potentially affecting the accuracy of reported prevalence rates. Although regression models were tested and results interpreted cautiously, multiple testing could increase the risk of Type I error. Finally, the cross-sectional design restricts our ability to infer causal relationships or assess changes in prevalence over time. Future research should employ longitudinal designs, explore diverse populations across different regions, and test targeted intervention strategies to better understand causal pathways and effective approaches for reducing self-stigma.

## Conclusions

This study underscores the significant burden of self-stigma and psychological distress among Saudi Arabian men, emphasizing the need to prioritize mental health as a public health concern. Our findings suggest that male-targeted anti-stigma campaigns in community and digital spaces, integrating mental health literacy into religious and educational institutions, and training primary care providers to address internalized stigma during consultations may be beneficial. Given the cultural homogeneity of the Saudi population and the widespread use of social media across all age groups, our results provide a valuable foundation for broader research and policy development, while recognizing that causal inferences cannot be drawn from a cross-sectional design. Future research involving broader and more diverse populations across different regions of Saudi Arabia is recommended to deepen the understanding of these issues. By contributing to the limited literature on male mental health in the Kingdom, this study offers insights to guide culturally appropriate, gender-sensitive interventions.

## Appendices

**Table 5:** Section 1: Sociodemographic Data

Q#	Question	Answer Choices
1	Age (in years)	Open response (e.g., 25)
2	Do you agree to participate in this questionnaire?	Yes / No
3	Gender	Male / Female
4	Data collector ID	Open response
5	Weight (kg)	Open response (e.g., 70)
6	Height (cm)	Open response (e.g., 160)
7	Monthly family income	Less than 5,000 SAR / 5,000–10,000 SAR / 10,000–15,000 SAR / More than 15,000 SAR
8	Nationality	Saudi / Non-Saudi
9	Administrative region of residence	Jazan Region / Other region (e.g., Riyadh, Makkah, Asir)
10	Place of residence	Village / City
11	Marital status	Married / Single / Divorced / Widowed
12	Number of family members	Open response
13	Are you a smoker?	Yes, I am a smoker / I am a former smoker / No, I am not a smoker
14	Type of smoking (if smoker, select most used)	Cigarettes / Shisha-Hookah / Electronic Hookah

**Table 6:** Section 2: DASS-21* ^*^[13] Instructions: Please read each statement and circle a number (0, 1, 2, or 3) that indicates how much the statement applied to you over the past week Response Options: 0 = Did not apply to me at all. 1 = Applied to me to some degree, or some of the time. 2 = Applied to me to a considerable degree, or a good part of time. 3 = Applied to me very much, or most of the time The DASS-21 is publicly accessible for non-commercial academic purposes DASS-21: Depression, Anxiety, and Stress Scale-21

Q#	Item
1	I found it hard to wind down
2	I was aware of dryness of my mouth
3	I couldn't seem to experience any positive feeling at all
4	I experienced breathing difficulty (e.g., excessively rapid breathing, breathlessness without exertion)
5	I found it difficult to work up the initiative to do things
6	I tended to over-react to situations
7	I experienced trembling (e.g., in the hands)
8	I felt that I was using a lot of nervous energy
9	I was worried about situations in which I might panic and make a fool of myself
10	I felt that I had nothing to look forward to
11	I found myself getting agitated
12	I found it difficult to relax
13	I felt down-hearted and blue
14	I was intolerant of anything that kept me from getting on with what I was doing
15	I felt I was close to panic
16	I was unable to become enthusiastic about anything
17	I felt I wasn't worth much as a person
18	I felt that I was rather touchy
19	I was aware of the action of my heart in the absence of exertion (e.g., increased rate, missed beat)
20	I felt scared without any good reason
21	I felt that life was meaningless

**Table 7:** Section 3: SSDS* ^*^[18,19] Instructions: The following statements are designed to assess the extent to which a person may hold stigmatizing attitudes towards themselves in relation to having depression. Please indicate your level of agreement with each item Response options (5-point Likert scale): 1 = Strongly Agree. 2 = Agree. 3 = Neutral. 4 = Disagree. 5 = Strongly Disagree. A higher score indicates greater self-stigma Permission to use the SSDS was obtained from the author of the validated Arabic translation SSDS: Self-Stigma of Depression Scale

Subscale	Q#	Item
Shame	1	I would feel ashamed.
	2	I would feel embarrassed.
	3	I would feel inferior to other people.
	4	I would feel disappointed in myself.
Self-blame	5	I would think I should be able to cope with things.
	6	I would think I should be able to “pull myself together.”
	7	I would think I should be stronger.
	8	I would think I only had myself to blame.
Help-seeking Inhibition	9	I would feel embarrassed about seeking professional help for depression.
	10	I would feel embarrassed if others knew I was seeking professional help for depression.
	11	I would see myself as weak if I took antidepressants.
	12	I wouldn’t want people to know that I wasn’t coping.
Social Inadequacy	13	I would feel I couldn’t contribute much socially.
	14	I would feel inadequate around other people.
	15	I would feel like I was good company. (Reverse-scored)
